# Supplementary material for: Effects of Physical Exercise on Cerebral Blood Velocity in Older Adults: A Systematic Review and Meta−Analysis
Source: Behav Sci (Basel). 2023 Oct 16;13(10):847. doi: 10.3390/bs13100847 (PMC10604216; doi:10.3390/bs13100847)
Supplement: Supplementary file 1 [file behavsci-13-00847-s001.zip › Table S2.pdf]

Table S2. Search strategies adapted to each platform.

Platform	Search strategy	Number of results
PubMed	(((((Transcranial doppler[Title/Abstract] OR transcranial doppler ultrasonography[Title/Abstract]) OR ultrasonography[Title/Abstract])) AND (((Cerebral blood flow[Title/Abstract]) OR cerebral circulation[Title/Abstract]) OR cerebral perfusion pressure[Title/Abstract])) AND (((Exercise[Title/Abstract]) OR physical activity[Title/Abstract]) OR training[Title/Abstract])) AND (((Elderly[Title/Abstract]) OR aged[Title/Abstract]) OR older people[Title/Abstract])	12
Web of Science	((((((TI="Transcranial doppler" OR AB="Transcranial doppler") OR (TI="transcranial doppler ultrasonography" OR AB="transcranial doppler ultrasonography")) OR (TI=ultrasonography OR AB=ultrasonography))) AND ((((TI="Cerebral blood flow" OR AB="Cerebral blood flow")) OR (TI="cerebral circulation" OR AB="cerebral circulation")) OR (TI="cerebral perfusion pressure" OR AB="cerebral perfusion pressure"))) AND ((((TI=Exercise OR AB=Exercise)) OR (TI="physical activity" OR AB="physical activity")) OR (TI=training OR AB=training))) AND ((((TI=Elderly OR AB=Elderly)) OR (TI=aged OR AB=aged)) OR (TI="older people" OR AB="older people"))	38
EBSCO	((((((TI "Transcranial doppler" OR AB "Transcranial doppler") OR (TI "transcranial doppler ultrasonography" OR AB "transcranial doppler ultrasonography")) OR (TI ultrasonography OR AB ultrasonography))) AND ((((TI "Cerebral blood flow" OR AB "Cerebral blood flow")) OR (TI "cerebral circulation" OR AB "cerebral circulation")) OR (TI "cerebral perfusion pressure" OR AB "cerebral perfusion pressure"))) AND ((((TI Exercise OR AB Exercise)) OR (TI "physical activity" OR AB "physical activity")) OR (TI training OR AB training))) AND ((((TI Elderly OR AB Elderly)) OR (TI aged OR AB aged)) OR (TI "older people" OR AB "older people"))	8
Scopus	(TITLE-ABS-KEY ("transcranial doppler" OR "transcranial doppler ultrasonography" OR ultrasonography) AND TITLE-ABS-KEY ("cerebral blood flow" OR "cerebral circulation" OR "cerebral perfusion pressure") AND TITLE-ABS-KEY (exercise OR "physical activity"OR training) AND TITLE-ABS-KEY (elderly OR aged OR "older people"))	100
ScienceDirect	("transcranial doppler" OR "transcranial doppler ultrasonography") AND ("cerebral blood flow" OR "cerebral circulation") AND (exercise OR "physical activity") AND (elderly OR aged)	329
