# Supplementary material for: Effects of Physical Exercise on Cerebral Blood Velocity in Older Adults: A Systematic Review and Meta−Analysis
Source: Behav Sci (Basel). 2023 Oct 16;13(10):847. doi: 10.3390/bs13100847 (PMC10604216; doi:10.3390/bs13100847)
Supplement: Supplementary file 1 [file behavsci-13-00847-s001.zip › Table S3.pdf]

Table S3. Certainty assessment using GRADEpro.

Certainty assessment							№ of patients		Effect		Certainty	Importance
№ of studies	Study design	Risk of bias	Inconsistency	Indirectness	Imprecision	Other considerations	Exercise	No exercise	Relative (95% CI)	Absolute (95% CI)		
**Cerebral blood velocity**												
4	randomized trials	not serious	not serious	not serious	serious	strong association	94	89	-	MD **3.58 cm/s higher** (0.51 higher to 6.65 higher)	⨁⨁⨁⨁ High	IMPORTANT

CI: confidence interval; **MD:** mean difference
