# Supplementary figures and images for: Effects of Physical Exercise on Cerebral Blood Velocity in Older Adults: A Systematic Review and Meta−Analysis
Source: Behav Sci (Basel). 2023 Oct 16;13(10):847. doi: 10.3390/bs13100847 (PMC10604216; doi:10.3390/bs13100847)

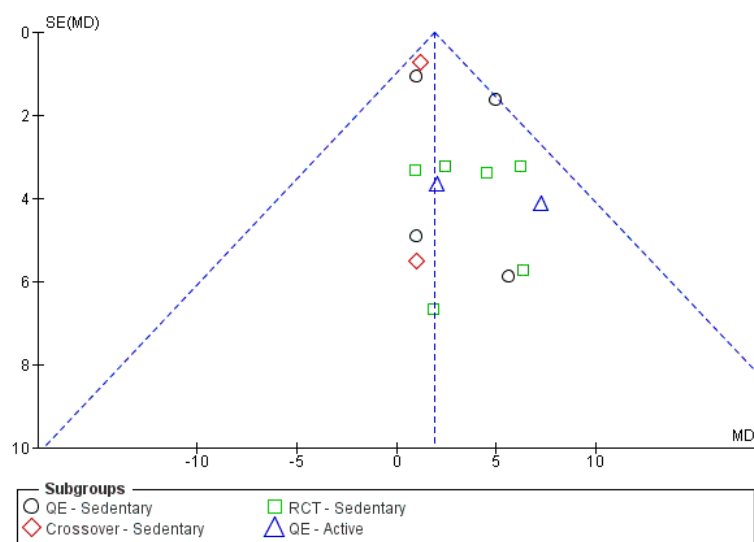

**Figure S1.** Funnel plot assessing potential publication bias.

Supplement: Supplementary file 1 [file behavsci-13-00847-s001.zip › Figure S1.pdf]
